# Supplementary material for: Comparison of physician-delivered models of virtual and home-based in-person care for adults in the last 90 days of life with cancer and terminal noncancer illness during the COVID-19 pandemic
Source: PLoS One. 2024 Nov 27;19(11):e0301813. doi: 10.1371/journal.pone.0301813 (PMC11602086; doi:10.1371/journal.pone.0301813)
Supplement: S2 Table — (DOCX) [file pone.0301813.s002.docx]

**S2 Table. List of virtual care fee codes according to pandemic time periods**

| **Pre-Pandemic Fee Codes** | |
| --- | --- |
| B099A | Tracking Code |
| B100A | First Telemedicine Patient Encounter premium |
| B101A | First Cancelled/Missed Telemedicine Patient Encounter premium |
| B102A | First Technical Difficulties Abandoned Patient Encounter premium |
| B200A | Subsequent Telemedicine Patient Encounter premium |
| B201A | Subsequent Missed/Cancelled Telemedicine Patient Encounter premium |
| B202A | Subsequent Technical Difficulties Abandoned Patient Encounter premium |
| G511A | Telephone management regarding a patient receiving palliative care at home |
| **Pandemic Fee Codes** | |
| K080A | Minor assessment of a patient by telephone or video or advice or information by telephone or video to a patient’s representative regarding health maintenance, diagnosis, treatment and/or prognosis. |
| K081A | a. Intermediate assessment of a patient by telephone or video, or advice or information by telephone or video to a patient’s representative regarding health maintenance, diagnosis, treatment and/or prognosis, if the service lasts a minimum of 10 minutes; or |
| b. Psychotherapy, psychiatric or primary mental health care, counselling or interview conducted by telephone or video, if the service lasts a minimum of 10 minutes. | |
| K082A | Psychotherapy, psychiatric or primary mental health care, counselling or interview conducted by telephone or video per unit (unit means half hour or major part thereof). |
| K083A | Specialist Consultations and Visits by telephone or video. |
| B203A | Synchronous video visits with a patient in the home or another location of their choice (i.e. the patient is not at a patient host site). |
| G511A | Telephone management regarding a patient receiving palliative care at home |
