# Supplementary material for: Comparison of physician-delivered models of virtual and home-based in-person care for adults in the last 90 days of life with cancer and terminal noncancer illness during the COVID-19 pandemic
Source: PLoS One. 2024 Nov 27;19(11):e0301813. doi: 10.1371/journal.pone.0301813 (PMC11602086; doi:10.1371/journal.pone.0301813)
Supplement: S3 Table — (DOCX) [file pone.0301813.s003.docx]

**S3 Table. Physician demographics (as of first end-of-life visit for people the physician saw in each disease cohort**)

| Characteristic | Cancer  N=18520 | Chronic Organ Failure  N=12476 | Dementia  N= 4550 | Multimorbidity  N=12438 | Standardized differences | | |
| --- | --- | --- | --- | --- | --- | --- | --- |
|  |  |  |  |  | **Cancer vs Chronic organ failure** | **Cancer vs Dementia** | **Cancer vs multimorbidity** |
| Sex, n% |  |  |  |  |  |  |  |
| Female | 7,914 (42.7%) | 5,007 (40.1%) | 1,823 (40.1%) | 5,006 (40.2%) | 0.05 | 0.05 | 0.05 |
| Male | 10,606 (57.3%) | 7,469 (59.9%) | 2,727 (59.9%) | 7,432 (59.8%) | 0.05 | 0.05 | 0.05 |
| Age, median (IQR) | 48 (38-59) | 49 (39-59) | 49 (39-60) | 49 (39-59) | 0.03 | 0.06 | 0.03 |
| Education |  |  |  |  |  |  |  |
| Canadian graduate | 9,484 (51.2%) | 6,301 (50.5%) | 2,246 (49.4%) | 6,413 (51.6%) | 0.01 | 0.04 | 0.01 |
| International graduate | 3,658 (19.8%) | 2,702 (21.7%) | 1,085 (23.8%) | 2,578 (20.7%) | 0.05 | 0.1 | 0.02 |
| Missing | 5,378 (29.0%) | 3,473 (27.8%) | 1,219 (26.8%) | 3,447 (27.7%) | 0.03 | 0.05 | 0.03 |
| Rural practice, n (%) | 1,036 (5.6%) | 730 (5.9%) | 217 (4.8%) | 688 (5.5%) | 0.01 | 0.04 | 0 |
| Years in practice, median (IQR) | 21 (11-33) | 22 (11-33) | 22 (11-33) | 22 (11-33) | 0.03 | 0.05 | 0.03 |
| Physician speciality - Family Medicine/General Practitioner, n(%) | 10,563 (57.0%) | 7,483 (60.0%) | 3,006 (66.1%) | 7,291 (58.6%) | 0.06 | 0.19 | 0.03 |
| Palliative Care Specialist | 732 (4.0%) | 622 (5.0%) | 386 (8.5%) | 651 (5.2%) | 0.05 | 0.19 | 0.06 |
| No of unique end-of-life visits in calendar year of index (tertiles), n(%) |  |  |  |  |  |  |  |
| 1 | 8,032 (43.4%) | 3,744 (30.0%) | 810 (17.8%) | 3,550 (28.5%) | 0.28 | 0.58 | 0.31 |
| 2 | 5,840 (31.5%) | 4,381 (35.1%) | 1,399 (30.7%) | 4,328 (34.8%) | 0.08 | 0.02 | 0.07 |
| 3 | 4,648 (25.1%) | 4,351 (34.9%) | 2,341 (51.5%) | 4,560 (36.7%) | 0.21 | 0.56 | 0.25 |
