# Supplementary material for: Comparison of physician-delivered models of virtual and home-based in-person care for adults in the last 90 days of life with cancer and terminal noncancer illness during the COVID-19 pandemic
Source: PLoS One. 2024 Nov 27;19(11):e0301813. doi: 10.1371/journal.pone.0301813 (PMC11602086; doi:10.1371/journal.pone.0301813)
Supplement: S4 Table — (DOCX) [file pone.0301813.s004.docx]

**S4 Table. Associations of receiving exclusively home-based in-person and mixed model of care in the last 90 days of life according to the type of serious illness using cancer as main referent group**

|  | Exclusively home-based in-person EOL visits,  n (%) | Mixed EOL visits,  n (%) | All home-based in-person EOL visits vs Mixed EOL visits | |
| --- | --- | --- | --- | --- |
|  |  |  | **Unadjusted OR (95% CI)** | **Adjusted OR (95% CI)** |
| Cancer | 1,951 (3.3%) | 23,124 (39.3%) | 1.0 (ref) | |
| Chronic organ failure | 495  (3.1%) | 3,927 (24.4%) | 1.49 (1.35, 1.66) | 1.45 (1.30, 1.62) |
| Dementia | 255  (6.5%) | 1,482 (37.9%) | 2.04 (1.77, 2.35) | 1.47 (1.27, 1.71) |
| Multimorbidity | 162  (3.1%) | 1,468 (28.0%) | 1.31 (1.10, 1.55) | 1.14 (0.95, 1.36) |

*Groups are not mutually exclusive (e.g., a dementia people could also be in the chronic organ failure group).

Models were adjusted for age, sex, ethnicity, comorbidities, rurality, neighbourhood income and hospital frailty risk score.

Abbreviation: OR = odds ratio
