# Supplementary material for: Comparison of physician-delivered models of virtual and home-based in-person care for adults in the last 90 days of life with cancer and terminal noncancer illness during the COVID-19 pandemic
Source: PLoS One. 2024 Nov 27;19(11):e0301813. doi: 10.1371/journal.pone.0301813 (PMC11602086; doi:10.1371/journal.pone.0301813)
Supplement: S5 Table — (DOCX) [file pone.0301813.s005.docx]

**S5 Table. Baseline characteristics of the study cohort according to models of care in the last 90 days of life**

| Characteristic | Exclusively virtual | Exclusively home-based in-person | Mixed virtual and home-based in-person | Standardized differences | | | | |  |
| --- | --- | --- | --- | --- | --- | --- | --- | --- | --- |
|  |  |  |  | Virtual vs Mixed | Home-based in-person vs Mixed | | Virtual vs Home-based in-person | | |
| Age, Mean ± SD | 75.70 ± 12.93 | 83.97 ± 11.60 | 78.14 ± 12.95 | 0.19 | 0.47 | | 0.67 | | |
| Sex, n% |  |  |  |  |  | |  | | |
| Female | 20,626 (44.9%) | 1,456 (57.6%) | 13,894 (50.6%) | 0.12 | 0.14 | | 0.26 | | |
| Male | 25,337 (55.1%) | 1,073 (42.4%) | 13,544 (49.4%) | 0.12 | 0.14 | | 0.26 | | |
| Neighbourhood income quintile |  |  |  |  |  | |  | | |
| 1 | 10,958 (23.8%) | 599 (23.7%) | 5,580 (20.3%) | 0.08 | 0.08 | | 0 | | |
| 2 | 10,243 (22.3%) | 540 (21.4%) | 5,763 (21.0%) | 0.03 | 0.01 | | 0.02 | | |
| 3 | 9,239 (20.1%) | 495 (19.6%) | 5,547 (20.2%) | 0 | 0.02 | | 0.01 | | |
| 4 | 7,829 (17.0%) | 434 (17.2%) | 5,103 (18.6%) | 0.04 | 0.04 | | 0 | | |
| 5 | 7,517 (16.4%) | 444 (17.6%) | 5,345 (19.5%) | 0.08 | 0.05 | | 0.03 | | |
| Missing information | 177 (0.4%) | 17 (0.7%) | 100 (0.4%) | 0 | 0.04 | | 0.04 | | |
| Rural residence |  |  |  |  |  | |  | | |
| N | 40,832 (88.8%) | 2,175 (86.0%) | 24,398 (88.9%) | 0 | 0.09 | | 0.09 | | |
| Y | 4,976 (10.8%) | 338 (13.4%) | 2,945 (10.7%) | 0 | 0.08 | | 0.08 | | |
| Missing information | 155 (0.3%) | 16 (0.6%) | 95 (0.3%) | 0 | 0.04 | | 0.04 | | |
| Ethnicity, n (%) |  |  |  |  |  | |  | | |
| Chinese | 1,465 (3.2%) | 44 (1.7%) | 767 (2.8%) | 0.02 | 0.07 | | 0.09 | | |
| General | 43,078 (93.7%) | 2,458 (97.2%) | 26,134 (95.2%) | 0.07 | 0.1 | | 0.17 | | |
| Missing information | <=5 (0.0%) | 0 (0.0%) | 6 (0.0%) | 0.01 | 0.02 | | 0.01 | | |
| Chronic Disease |  |  |  |  |  |  | |  |  |
| Cancer | 33,819 (73.6%) | 1,951 (77.1%) | 23,124 (84.3%) | 0.26 | 0.18 | 0.08 | |  |  |
| Heart failure | 14,648 (31.9%) | 836 (33.1%) | 7,651 (27.9%) | 0.09 | 0.11 | 0.03 | |  |  |
| COPD | 10,856 (23.6%) | 580 (22.9%) |  | 0.07 | 0.06 | 0.02 | |  |  |
| Dementia | 6,390 (13.9%) | 808 (31.9%) | 5,088 (18.5%) | 0.13 | 0.31 | 0.44 | |  |  |
| Severe liver disease | 778 (1.7%) | 37 (1.5%) | 543 (2.0%) | 0.02 | 0.04 | 0.02 | |  |  |
| Diabetes | 21,181 (46.1%) | 914 (36.1%) | 10,428 (38.0%) | 0.16 | 0.04 | 0.2 | |  |  |
| Hypertension | 36,359 (79.1%) | 2,025 (80.1%) | 20,807 (75.8%) | 0.08 | 0.1 | 0.02 | |  |  |
| End-stage renal disease | 14,347 (31.2%) | 672 (26.6%) | 7,228 (26.3%) | 0.11 | 0.01 | 0.1 | |  |  |
| Stroke | 5,204 (11.3%) | 403 (15.9%) | 3,259 (11.9%) | 0.02 | 0.12 | 0.13 | |  |  |
| Psychotic disorder | 690 (1.5%) | 40 (1.6%) | 274 (1.0%) | 0.05 | 0.05 | 0.01 | |  |  |
| Non-psychotic disorder | 11,739 (25.5%) | 629 (24.9%) | 6,858 (25.0%) | 0.01 | 0 | 0.02 | |  |  |
| Alcohol and substance use disorder | 1,865 (4.1%) | 57 (2.3%) | 551 (2.0%) | 0.12 | 0.02 | 0.1 | |  |  |
| Hospital frailty risk score |  |  |  |  |  |  | |  |  |
| 0. 0 | 5,704 (12.4%) | 196 (7.8%) | 3,699 (13.5%) | 0.03 | 0.19 | 0.16 | |  |  |
| 0.1 - 4.9 | 10,902 (23.7%) | 527 (20.8%) | 6,862 (25.0%) | 0.03 | 0.1 | 0.07 | |  |  |
| 5.0 - 8.9 | 5,908 (12.9%) | 389 (15.4%) | 3,809 (13.9%) | 0.03 | 0.04 | 0.07 | |  |  |
| 9.0 + | 9,561 (20.8%) | 812 (32.1%) | 6,300 (23.0%) | 0.05 | 0.21 | 0.26 | |  |  |
| No hospitalizations | 13,888 (30.2%) | 605 (23.9%) | 6,768 (24.7%) | 0.12 | 0.02 | 0.14 | |  |  |
