# Supplementary material for: Comparison of physician-delivered models of virtual and home-based in-person care for adults in the last 90 days of life with cancer and terminal noncancer illness during the COVID-19 pandemic
Source: PLoS One. 2024 Nov 27;19(11):e0301813. doi: 10.1371/journal.pone.0301813 (PMC11602086; doi:10.1371/journal.pone.0301813)
Supplement: Attachment — Submitted filename: Virtual EOL Care Response Letter FEB13-2024.docx [file pone.0301813.s006.docx]

Kartikeya Rajdev, MD

Academic Editor

PLOS ONE

**PONE-D-23-35922 - "Comparison of physician-delivered models of virtual and home-based in-person care for adults in the last 90 days of life with cancer and terminal noncancer illness during the COVID-19 pandemic”**

Thank you for providing us the opportunity to resubmit our work to *PLOS One*. We are pleased that the editors and reviewers found our study to be important, valuable, relevant, and of considerable interest. Their helpful suggestions and comments have helped us to improve the quality of the manuscript. We have addressed their concerns in detail below.

The following is a summary of the extensive changes we made to the manuscript in response to yours and the reviewer’s suggestions:

1) Provided clear and actionable insights to enhance the study’s relevance for policymakers.
2) Updated Table 2 to report the total number and proportion of people receiving exclusively in-person care.
3) Modified the introduction to improve transition to objective statement.
4) Highlighted the principal knowledge gaps in the introduction.
5) Provided additional rationale in multiple areas in the methods, such as for the exclusion of individuals from the study cohort.
6) Enhanced the interpretation of the main findings through recognition of residual confounding.
7) Updated the limitations to recognize lack of measures on perceptions of quality of care.
8) Reduced repetitive language throughout the manuscript to improve readability and reduce overall length.

**EDITOR COMMENTS:**

1. **Please ensure that your manuscript meets PLOS ONE's style requirements, including those for file naming.**

We updated the manuscript as per PLOS ONE's style requirements.

2. **Please state what role the funders took in the study. If the funders had no role, please state: "The funders had no role in study design, data collection and analysis, decision to publish, or preparation of the manuscript."**

We added the suggested statement to the online financial disclosure form: “The funders had no role in study design, data collection and analysis, decision to publish, or preparation of the manuscript.”

3. **All PLOS journals now require all data underlying the findings described in their manuscript to be freely available to other researchers, either a. In a public repository, b. Within the manuscript itself, or c. Uploaded as supplementary information. This policy applies to all data except where public deposition would breach compliance with the protocol approved by your research ethics board. If your data cannot be made publicly available for ethical or legal reasons (e.g., public availability would compromise patient privacy), please explain your reasons on resubmission and your exemption request will be escalated for approval.**

We request an exemption from this policy as legal data sharing agreements prohibit ICES from making the dataset publicly available; access may be granted to those who meet pre-specified criteria for confidential access, available at www.ices.on.ca/DAS.

We previously indicated these restrictions in the manuscript as follows,

“The dataset from this study is held securely in coded form at ICES. While data sharing agreements prohibit ICES from making the dataset publicly available, access may be granted to those who meet pre-specified criteria for confidential access, available at www.ices.on.ca/DAS. The full dataset creation plan and underlying analytic code are available from the authors upon request, understanding that the computer programs may rely upon coding templates or macros that are unique to ICES and are therefore either inaccessible or may require modification.” **(Data Sharing, page 20, lines 312-317)**

**REVIEWERS’ COMMENTS:**

**Reviewer #1**

1. **Very well-designed study. In table 2, I couldn’t find data regarding in person care, maybe I missed it - can you include it.**

We updated Table 2 to report the total number and proportion of people receiving exclusively in-person care as requested. **(Table 2, page 14)** We had originally included these data in Supplementary Table 2 because the main comparison of interest was the receipt of exclusively virtual versus mixed models of care.

2. **Was there any change in perception by patients and families regarding quality of care in these models?**

We did not measure patient or family perceptions regarding quality of care as these measures are not available in administrative health data in Ontario. We updated the limitations to recognize this important point as follows,

“Second, the administrative databases that we used do not measure a person's individual care needs, preferences, or perceived quality of care, which may direct the model of care they received in their last 90 days of life.” Prior complimentary qualitative research conducted by our team through interviews with patients, caregivers, and clinicians identified preferences toward use of a mixed model of care tailored to the preferences and needs of the individual. **(Strengths and Limitations, page 19, lines 287-290)**

**Reviewer #2**

1. **Several statements were repeated multiple time throughout the article. If possible, to try to minimize the repetitions. Sample size is great. Lot of statistics related calculations had to be used. Appreciate the work from the authors. I liked the tabular format for displaying the statistics. Reducing the repetitions will trim the article size and make it more interesting for readers.**

Thank you for the suggestion. We reviewed the manuscript and reduced repetitive language throughout to improve readability and reduce overall length.

**Reviewer #3**

1. **Introduction: The introduction of the article provides a solid foundation but lacks a smooth transition to the study objective. A more seamless connection between the background information and the specific aim would enhance reader comprehension.**

We modified the introduction as follows,

“Despite observed differences in EOL care delivery models between people with different types of serious illness before the pandemic, it remains unknown if these differences persisted in the pandemic during a period of widespread virtual care use. Further, it is unknown if a preferential model of mixed model of care is used more frequently among people with cancer than among people with terminal noncancer illness.

To address these existing knowledge gaps in patterns of end-of-life care delivery during the pandemic, we measured the association between types of serious illness (cancer and noncancer terminal illness: chronic organ failure, dementia, multimorbidity) and the use of different physician-delivered care models (exclusively virtual, exclusively home-based in-person, and mixed model of care) near the EOL.” **(Introduction, page 3-4, lines 45-53)**

2. **Introduction: Additionally, explicitly highlighting the research gap or unknown aspects that the study seeks to address could enhance the introduction's impact, engaging readers and underscoring the study's significance.**

We modified the introduction as follows,

“Despite observed differences in EOL care delivery models between people with different types of serious illness before the pandemic, it remains unknown if these differences persisted in the pandemic during a period of widespread virtual care use. Further, it is unknown if a preferential model of mixed model of care is used more frequently among people with cancer than among people with terminal noncancer illness.” **(Introduction, page 3-4, lines 45-48)**

3. **Methods (Compliance with Reporting Guidelines): The article demonstrates clear reporting in adherence to guidelines. However, a brief explanation of the rationale for choosing linked clinical and administrative datasets would offer readers a deeper understanding of the study design's appropriateness. Including such context could improve the overall transparency of the research.**

We added to the methods the following rationale,

“The use of population-level linked administrative data minimizes selection bias and improves the diversity of the study cohort to strengthen the overall generalizability of the study and its principal findings.” **(Methods, page 5, lines 65-67)**

We previously stated the strengths of using population-level linked clinical and administrative datasets in the discussion as follows,

“The study's major strength lies in its population-level coverage. The inclusion of a diverse range of participants from a large population ensures the findings represent the population as a whole, enhancing the study's generalizability and minimizing selection bias.” **(Strengths and Limitations, page 18, lines 276-278)**

4. **Methods (Exclusion Criteria and Rationale): The exclusion criteria are well-defined, but the rationale behind each criterion could be more explicitly stated. Providing a clearer justification, such as explaining why individuals with fewer than two visits were excluded, would bolster the methodological rigor, and strengthen the study's foundation.**

We added the following clarification and rationale,

“We excluded adults 1) whose last 90 days of life started before March 14, 2020, which preceded the onset of the pandemic and the majority of resulting health system changes;…” **(Methods, page 6, lines 83-84)**

“We also excluded individuals who were non-Ontario residents, ineligible for OHIP for more than 90 days continuously in the prior year and therefore could not receive publicly insured health services, and people who did not access the Ontario healthcare system at least once in the past 10 years to ensure people are still residing in the province. **(Methods, page 6, lines 90-94)**

We provided rationale for the exclusion of individuals with fewer than two visits in our original manuscript as follows,

“The need to have 2 or more visits was necessary to determine if people received exclusively in-person, exclusively virtual, or mixed models of physician-delivered care in the last 90 days of life (the primary outcome).” **(Methods, page 6, lines 88-90)**

We provided rationale for the remaining exclusion criteria in our original manuscript as follows,

“…2) without prevalent cancer, chronic organ failure, or dementia at 90 days before death (the primary exposures); 3) residents living in nursing homes in the past two years as care delivery during the pandemic was significantly interrupted in this setting; and 4) with ≤1 visit or who were institutionalized during the last 90 days of life. The need to have 2 or more visits was necessary to determine if people received exclusively in-person, exclusively virtual, or mixed models of physician-delivered care in the last 90 days of life (the primary outcome).” **(Methods, page 6, lines 84-90)**

5. **Results: While the article adjusts for potential confounding variables in the logistic regression models, a brief acknowledgment and discussion of limitations or potential confounders related to these adjustments would enhance the statistical validity of the findings. Addressing this aspect would provide a more comprehensive understanding for readers, ensuring a more robust interpretation of the results.**

We added the following to the interpretation of the principal study findings,

“Although we adjusted our statistical models for multiple potential confounders, the observed differences may also reflect residual confounding due to unmeasured factors such as the effects of the pandemic on access to healthcare and its delivery or individual patient preferences for specific care modalities.” **(Discussion, page 16, lines 229-233)**

6. **Discussion: In the discussion section, there is a mention of implications for healthcare planning, but the article lacks specific recommendations or strategies based on observed differences. Adding actionable insights would enhance the article's relevance for policymakers, ensuring that the research findings are not only informative but also practical in guiding future healthcare planning efforts. Providing concrete suggestions would bridge the gap between research outcomes and practical application, making the discussion more impactful.**

We modified the discussion of the application of our findings as follows,

“Furthermore, our findings inform future healthcare resource planning to accommodate different delivery models based on an individual's chronic illness. For example, governments may need to increase and provide additional home care resources to deliver care to patients living with dementia in their homes. These additional supports will require financial investments and policies on supporting reimbursement from payers. In contrast, there may be a need for additional investment in virtual care near the EOL for heart failure patients, ensuring that reimbursement is provided at 100%, among other tailored strategies. This is particularly relevant given the widespread increase in use of virtual care that occurred during the pandemic with recent modifications to reimbursement policies provided by governments.” **(Discussion, page 16-17, lines 234-243)**
